# Cruise traveling behavior post-COVID-19: An integrated model of health protection motivation, travel constraint and social learning

**DOI:** 10.3389/fpsyg.2022.949288

**Published:** 2022-07-22

**Authors:** Kum Fai Yuen, Lanhui Cai, Xueqin Wang

**Affiliations:** ^1^School of Civil and Environmental Engineering, Nanyang Technological University, Singapore, Singapore; ^2^Department of International Logistics, Chung-Ang University, Seoul, South Korea

**Keywords:** cruise travel intention, health protection, social learning, travel constraint, post-COVID-19

## Abstract

Travel restrictions have harmed the cruise industry as a result of the COVID-19 pandemic. The goal of this study, which is attributed to permanent changes in the regulatory and social landscape, is to identify and examine the factors influencing post-COVID-19 cruise travel intention. To explain cruise travel intention, we developed a theoretical model incorporating health protection motivation, social learning and travel constraint theories. An online survey was conducted with 400 valid responses collected from Singapore. The theoretical model was estimated using structural equation modeling and the survey data. The findings indicate that (1) observing societal behavior, (2) observing the COVID-19 situation, (3) threat appraisal, and (4) coping appraisal all have a direct influence on travel constraint negotiation. Furthermore, travel constraint negotiation and societal observation have a direct impact on intention. An intriguing finding is that observing the COVID-19 situation has no effect on threat assessment, which can be attributed to pandemic fatigue. The findings allow for a set of recommendations to cruise companies and policymakers for post-COVID-19 cruise industry management.

## Introduction

The emergence of coronavirus disease 2019 (COVID-19) in 2020 resulted in a pandemic, wreaking havoc on the world's medical and economic systems. To contain the spread of COVID-19, many countries closed their ports and borders, severely affecting the global tourism industry (Behsudi, 2020). Due to such travel restrictions, the cruise industry, which is part of the tourism industry, is also impacted (Tay, 2020). In the first half of 2020, multiple outbreaks occurred in cruises (Forster, 2020; Webeck, 2020) as a result of the virus spreading faster in crowded and semi-enclosed environments. This had a significant impact on consumers' perspectives of the cruise industry and their trust in cruise companies to manage the virus. Due to restrictions and the fear of contracting the virus, consumers actively avoided cruise travel (Tay, 2020). Carnival Corporation & PLC, the world's largest cruise company, reported an 85% decrease in revenue in the second quarter of 2020 compared to the same period in 2019, resulting in a GAAP loss of US$4.4 billion, compared to a net income of US$450 million in 2019 (Frizzell, 2020). Such losses are not unique, as many other cruise lines reported significant losses too (Norwegian Cruise Line Holdings Ltd, 2020; Royal Caribbean Group, 2020).

While experts predicted that the pandemic will last for years, post-COVID-19 is typically defined as an endemic, with the virus not disappearing but reappearing at different times and with different variants (Achenbach and Abutaleb, 2021; Charumilind et al., 2021; Lai, 2021; The Business Times, 2021). In this post-COVID-19 world, the cruise industry must adapt. Countries with better pandemic control experimented with cruise to nowhere in the latter half of 2020 (Teh, 2020; Hochberg, 2021; Wainwright, 2021), reducing losses. Countries began relaxing COVID-19 restrictions in 2021, but they required vaccination to entry certain areas (Emma et al., 2021; Hubler, 2021; Tan, 2021). Cruise lines are no exception, with similar regulations in place to better manage outbreak risk onboard (Espiner, 2021; Hines, 2021). These measures are in response to the constantly changing COVID-19 situation. Studies established that the pandemic altered consumer attitudes toward travel and cruising (Fennell, 2017; Holland et al., 2021; Pan et al., 2021; Zheng et al., 2021), requiring cruise companies to develop new and relevant strategies and implementations with COVID-19 in mind. However, whether consumers will resume cruise travel remains unclear. Thus, the objective of this study is to investigate the potential factors that will influence consumer intention to cruise travel.

There is currently limited research on the impact of the COVID-19 pandemic on consumer intention to cruise travel. Holland et al. (2021) explored the effects of COVID-19 on consumers' risk perceptions and intentions on cruise travel. In the research of Pan et al. (2021), consumer intention to cruise was explained through the integration of travel constraint theory and perspective theory. Using trust theory and risk management research, Quintal et al. (2022) analyzed consumer intentions to take cruises. All these papers attempt to explore the impact of the pandemic on consumer intentions to cruise; however, they all focus on the impact of consumer-perceived risks and restrictions posed by the pandemic on behavioral intentions. There are still some gaps that have not been addressed in previous studies.

First, past studies have neglected consumer coping capabilities and response efficacy. There is no doubt that the growing fear of contracting the virus is the primary cause of a shift in mindset and consumer priorities (Tay, 2020; Zheng et al., 2021). Following outbreaks on cruise lines, such as Diamond Princess and Carnival Cruises (Forster, 2020; Chen Lin, 2021; Yeginsu, 2021), consumers may believe that cruising activities came with the risk of early vacation termination, isolation onboard, contracting the virus, and even death. However, when individuals face risks, they will automatically stimulate the self-protective instinct of human beings and take countermeasures. Taking effective countermeasures can reduce the perception of danger and risk to a certain degree, increasing consumer behavior intention. According to protection motivation theory (PMT), which is commonly used for health-related issues or crises, when faced with a threat, people change their behavior to protect themselves (Rogers, 1975; Prasetyo et al., 2020; Zheng et al., 2021). PMT further asserts that whether individuals take recommended actions to protect themselves depends on their assessment of the threat level of the incident (threat appraisal) and the effectiveness of the recommended action (coping appraisal). Tourism studies have also used PMT to investigate how this fear leads to actions to protect themselves (Luo and Lam, 2020). Therefore, this study introduces the PMT to assess consumers' coping capabilities in the face of risk or threat.

Besides, previous research seems to fail to recognize the impact of social learning on an individual's traveling behavior. Individuals are influenced not only by their own fear of the pandemic, but also by the actions of others (Taylor, 2021). Ananian-Welsh and Williams (2014) claimed that the opposite is also true, where fear becomes normalized after observing societal attitudes toward an issue. As such, this study employs social learning theory (SLT) to address the gap in the social aspect and its impact on the public's cruising behavior (Bandura, 1999; Akers and Jennings, 2015).

Other research suggests applying travel constraint negotiation theory (TCNT), one commonly used theory to explain consumer travel behavior. People adapt and change their travel plans to work around the negotiations caused by COVID-19 (Jackson et al., 1993; Karl et al., 2021). Although PMT focuses on restrictions and how an individual acts to reduce the threat's risk or to mitigate the threat's negative impact, TCNT focuses on changing the individual's behavior and mindset to match the situation to their expectations. Moreover, individuals can travel as planned, but their expectations may not be met. As a result, to make the decision to travel, negotiations must take place.

Based on PMT, TCNT, and SLT, a comprehensive model integrating the potential factors that will influence consumer behavioral intentions in the COVID-19 context is presented in this study. Thereby, this study aims to fill a knowledge gap by investigating how factual observation of the COVID-19 situation, public behavior observation and travel constraint negotiation will ultimately affect an individual's intention to cruise travel post-COVID-19. This research would first delve deeper into the concepts of PMT and TCNT, followed by a brief examination of societal behavior using SLT.

Furthermore, this research is beneficial to stakeholders, such as policymakers and cruise companies. If the COVID-19 situation worsens and restrictions must be tightened, policymakers will be better able to formulate policies that have the least impact on stakeholders if they understand the importance of negotiation in consumer psychology. Similarly, by incorporating health protection tactics and communication, cruise lines will identify key weaknesses in their marketing strategy.

The remainder of the study is organized as follows. Firstly, a model will be developed by combining the concepts of SLT, PMT and TCNT to explain the relationships between protection motivation, societal influence, and negotiations on travel intentions. Subsequently, the survey and analytical methods will be developed. The results will then be organized and discussed. Finally, conclusions are reached. The implications and future research recommendations for stakeholders will be elaborated.

## Literature review

### Protection motivation theory

PMT has traditionally been used in personal health studies to explain an individual's psychology when confronted with a threat (Floyd et al., 2000; Yuen et al., 2020). One would be motivated to protect themselves, resulting in a change in behavior (Pan et al., 2022). PMT is used in a variety of studies, the majority of which deal with health risks (Yang et al., 2022). COVID-19 is the current greatest health risk. As a result, many studies, such as workplace risk, risk perceptions in retail settings, and social distancing management with COVID-19, employ PMT in their models (Zhang et al., 2021). Similarly, this study investigates the COVID-19 health threat that individuals face and how they would protect themselves against it. This study employs PMT to better understand the COVID-19 protection motivations that influence an individual's decision to cruise.

According to PMT, protection motivations are determined by individuals' threat appraisal and coping appraisal. Threat appraisal involves the evaluation of two dimensions: severity and vulnerability. Severity focuses on an individual's perception of the seriousness of a threat, while vulnerability is how sensitive and vulnerable an individual is to a threat. Therefore, threat appraisal is an individual's subjective evaluation of the overall risk. Coping appraisal reflects an individual's assessment of self-efficacy, response efficacy and response cost. Specifically, self-efficacy refers to their ability to carry out such actions; response efficacy is the effectiveness of their response to the threat; response cost is the cost of the action (Rogers, 1975; Floyd et al., 2000; Prentice-Dunn et al., 2009). The perceived ability to deal with COVID-19 is defined as the coping appraisal. The concept of threat appraisal and coping appraisal is widely used in studies of behavioral or psychological changes associated with COVID-19. Finally, behavioral intention is defined as the desire to engage in the behavior (Rogers, 1975; Floyd et al., 2000; Prentice-Dunn et al., 2009). PMT is applicable in this study's context because COVID-19 is a pandemic and cruise travel after COVID-19 would still pose a significant health risk. This raises the threat level because COVID-19 would cause severe contagious health problems. Furthermore, more preventive measures, such as masks, are required, which may affect individuals' coping appraisal.

### Travel constraint negotiation theory

TCNT is another relevant theory. It is concerned with an individual's negotiation of a situation in the face of constraints. TCNT is a theory frequently involved in research on leisure and travel behavior, which asserts that the relationship between tourists' travel preferences and actual actions will be intervened by travel restrictions. According to Karl et al. (2021), constraints are factors that reduce the ability, preference, or willingness to travel as planned (Crawford and Godbey, 1987; Crawford et al., 1991). It may be structural constraints (i.e., financial and time constraints), intra-personal constraints (i.e., health issues) and interpersonal constraints (i.e., lack of partners). In the case of travel behavior restrictions, individuals need to negotiate to adjust their preferences or behavior. The purpose of negotiation is to reduce the impact of travel restrictions on individual travel preferences.

Constraint negotiation theories, such as festival event constraints (Boo et al., 2014) and traveling with physical disabilities, have generally been used in travel research (Daniels et al., 2005). In addition, in the context of the COVID-19 pandemic, Yang et al. (2021) explained the consumers' travel intention by integrating constraint negotiation theory and theory of planed behavior. Karl et al. (2021) used an extended TCNT model to explore the relationship between restraint, negotiation, and travel behavior. In this study, travel constraints that could influence travel constraint negotiation are protection motivation factors (i.e., threat and coping appraisal).

### Social learning theory

PMT and TCNT assume that individuals are self-sufficient and do not consider the social factor in decision-making. Bandura's SLT is used to address how social learning influences intentions. SLT is used in a variety of fields of study, including criminal behavior studies (Akers and Jennings, 2015) and developmental psychology (Thelen et al., 1977).

Bandura (1999) asserts that, in addition to internal beliefs and motivations, external factors such as social learning affect the individual. Individuals may learn from other people's behaviors when exposed to a social environment. Their behavioral intention is influenced if they can replicate it when combined with positive or negative motivations (Bandura, 1999). This implies that by observing society's actions, individuals can learn and mimic socially acceptable behaviors. Thus, SLT is used in this study to understand how individuals' observations of society's behavior affect their decision to cruise in the post-COVID-19 period. SLT is used as a supporting theory in this study to show how social learning influences decision-making.

We distinguish between factual information and subjective information obtained from learning or observing society. “Observation of situation” is defined as factual information obtained from the situation that has not been altered by the actions and reactions of society. Statistics, news, and laws are some examples. Meanwhile, “observation of society” is defined as anecdotes of behaviors and subjective trends of similar behaviors. This is related to the social learning aspect. According to Floyd et al. (2000), environmental information influences protection motivation. This is similar to situation observation, in that factual information obtained from society influences the mindset of an individual's motivations to protect themselves.

### Theoretical model and arguments

This study employs psychology and sociology theories to investigate how protection motivation, travel constraint negotiation and social learning influence cruising intention. Table 1 summarizes the basic assumptions, key constructs and applications of the theories.

**Table 1:** Applied theories.

**Theory characteristics**	**Protection motivation theory**	**Travel constraint negotiation theory**	**Social learning theory**
Paradigm	Psychology	Psychology	Sociology
Basic assumption	Assumes that the protection against the threat is dependent on the motivation of an individual.	Assumes that negotiation process undergone by the individuals are similar and will explain the general psychological process.	Assumes individuals learn behaviors through observation.
Representative Constructs	Threat appraisal, coping appraisal	Travel constraint negotiation	Observation of society, observation of situation
Application to model	This theory illustrates consumer psychology when faced with a threat	This theory explains how the individual with travel constraints will negotiate to lower their expectations for travel to occur.	This theory explains how the observation of the society and situation affects one's intention.


When public health crises hit, people adopt protective motivations and coping strategies. Consumers' motivations to protect themselves from health threats can impact their cruise travel behavior in the context of the COVID-19 pandemic. Thus, PMT is the foundation. However, the factors involved in PMT are mainly the evaluation of consumers' internal factors, such as consumers' assessment of threats and consumers' self-efficacy in responding to threats, ignoring the social factors. As a social being, the individual's observation of the surrounding environment and the people around him/her affect their behavior. To this end, this study introduces SLT to conceptualize the social factors influencing consumers' cruise travel behavioral intentions.

**Figure 1:**
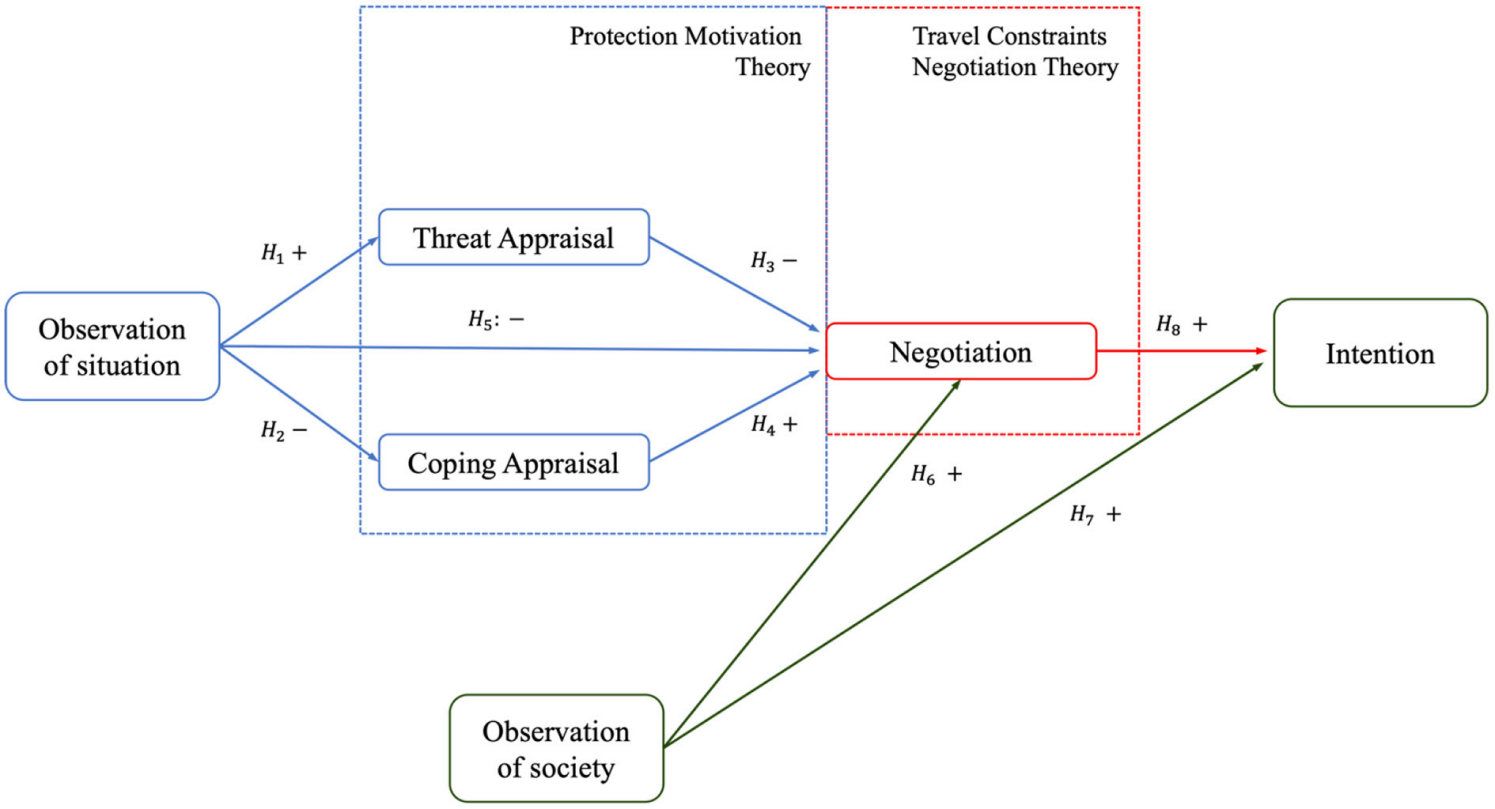
Proposed theoretical model.

Furthermore, a review of travel-related literature reveals that travel preferences or behavioral intention may be constrained by multiple factors (health and time). The threat of a pandemic and health concerns exacerbate the negative impact of these factors on travel behavior. As noted by TCNT, the influence of constraints on behavior intentions is adjusted by negotiation. Therefore, this study further introduces the negotiation structure in TCNT to explain the impact of the constraints imposed by the pandemic on travel-related behaviors. Therefore, this research proposes a theoretical model by integrating PMT, SLT and TCNT. Figure 1 depicts a theoretical model proposed using the theories from Table 1. The model depicts the relationship between the TNCT, PMT and SLT. Table 2 lists all the hypotheses involved in the proposed model.

**Table 2:** Hypotheses list.

**No**.	**Hypotheses**
H1.	Observation of the COVID-19 situation severity is positively associated with the individual's threat appraisal.
H2.	Observation of COVID-19 situation severity is negatively associated with the individual's coping appraisal.
H3.	Threat appraisal is negatively related to the individual's negotiation of cruise travel.
H4.	Coping appraisal is positively associated with the individual's negotiation toward cruise travel.
H5.	Observation of COVID-19 situation severity is negatively associated with the individual's negotiation toward cruise travel.
H6.	Observation of society's behavior toward cruise travel is positively associated with the individual's negotiation toward cruise travel.
H7.	Observation of society's behavior toward cruise travel is positively associated with the cruise travel behavioral intention.
H8.	Observation of society's behavior toward cruise travel is positively associated with the cruise travel behavioral intention.

### Hypotheses development

#### Protection motivation

According to PMT, when an individual observed the threat situation, he or she will appraise the level of threat posed to him or her. Individuals assess the threat's likelihood (threat vulnerability) and the extent of damage it can cause (threat severity) (Rogers, 1983; Wang et al., 2020). Fisher et al. (2018) applied PMT to cruise travel in a similar context. Hence, it is expected that after observing the COVID-19 situation, one would conduct threat assessment based on the severity and susceptibility of contracting the virus. If the COVID-19 situation worsens, the individual will consider it a high risk.

H1. Observation of the COVID-19 situation severity is positively associated with the individual's threat appraisal.

Individuals are motivated to protect themselves from harm after witnessing the threat situation. They assess the effectiveness of their response to the threat (response efficacy), their ability to carry out such actions (self-efficacy) and the cost of the action (response cost) (Rogers, 1983; Prentice-Dunn et al., 2009).

Threat situation observation is hypothesized to impact coping appraisal negatively. When the news reported that multiple cruises were “Petri-dishes” for COVID-19 cases, consumers became more concerned about contracting the virus. Because cruise passengers are generally risk-averse (Tarlow, 2006), they believe that being on a cruise increases their exposure to COVID-19. Hence, they would take extra precautions to protect themselves on board or avoid cruising altogether (Holland et al., 2021). As a result, a negative assessment of the COVID-19 situation will necessitate a higher cost of action or impair individuals' ability to deal with the threat.

H2. Observation of COVID-19 situation severity is negatively associated with the individual's coping appraisal.

#### Travel constraint negotiation

PMT investigated the individual's threat protection behavior. However, the protection behavior affects the travel appeal, which can be a constraint. The constraint causes negative emotions and a perceived difference between the positive benefits of traveling without restrictions and the actual benefits of the travel experience under health restrictions. Negotiation strategies are required to overcome the constraints that prevent intention from occurring. Negotiations strategies include managing their negative emotions and adjusting their travel expectations or actual travel behavior to patch the cognitive dissonance (Festinger, 1957; Karl et al., 2021).

In this study, an increase in COVID-19 threat appraisal causes more fear and negative emotions. Because consumers will be less willing to travel because they are afraid of infection and death, they will exert less effort in negotiating the constraints, and thus, threat appraisal negatively impacts negotiation (Zheng et al., 2021).

H3. Threat appraisal is negatively related to the individual's negotiation of cruise travel.

COVID-19 also causes a decrease in coping appraisal, which is reflected in more restrictions. Consumers must manage their expectations even more carefully. Online travel articles have promoted strategies for dealing with the COVID-19 restrictions (Endo, 2021; Wood, 2021). According to Kazeminia et al. (2015), larger constraints necessitate more negotiation until the constraints become too extreme, prohibiting any negotiations. Similarly, the following hypothesis is proposed:

H4. Coping appraisal is positively associated with the individual's negotiation toward cruise travel.

It is hypothesized that situation observation has a negative impact on negotiation, with threat appraisal and coping appraisal having an indirect effect, acting as mediators. The COVID-19 situation creates additional barriers to negotiation. According to Boo et al. (2014), the most significant constraint affecting negotiations is structural constraints, of which COVID-19 is a part. As a result, witnessing a more severe COVID-19 situation negatively affects their ability to cope with the difference. Such barriers required more energy to overcome, resulting in fewer negotiation attempts.

H5. Observation of COVID-19 situation severity is negatively associated with the individual's negotiation toward cruise travel.

#### Social learning

Following that, the social learning aspect is incorporated into the proposed model. People do not always learn through direct experience. Observational learning is also important in learning (Bandura, 1999). It is hypothesized that observation of society influences negotiation. As previously stated, cruise passengers are generally risk-averse, and thus, some are unwilling to take health risks for cruising activities. However, observations of more people boarding cruise ships may signal a possible exaggeration of the actual threat, influencing their decision to travel by cruise ship. This type of negotiation that occurs through social learning is referred to as normalization by Ananian-Welsh and Williams (2014). Individuals learn to accept it as a socially accepted norm through cognitive strategies.

H6. Observation of society's behavior toward cruise travel is positively associated with the individual's negotiation toward cruise travel.

Negotiation could also be viewed as a bridge between societal observation and an individual's behavioral intention. We can hypothesize that when an individual observes a societal behavior, it becomes a social push, and the individual is more likely to replicate similar behavior without directly evaluating the situation. This can be seen in panic buying, where people do not initially perceive a threat but are prone to panic buying when they recall others doing so (Taylor, 2021). As a result, increased observation of society's behavior may lead to increased behavioral intention, with negotiation acting as a mediator.

H7. Observation of society's behavior toward cruise travel is positively associated with the cruise travel behavioral intention.

Finally, the primary goal of the negotiation is to bridge the gap between individual expectations and actual action (Kazeminia et al., 2015; Karl et al., 2021). Negotiations allow people to adjust while still adhering to the constraints in place, allowing them to continue with their behavior. For behavioral intention to occur, this process requires either cognitive or behavioral adjustments. In this study, travel constraint negotiation would allow cruise travel during the post-COVID-19 period, despite the virus-imposed constraints.

H8. The individual's negotiation toward cruise travel is positively associated with the cruise travel behavioral intention.

## Methodology

### Measures and survey design

A survey was conducted to collect data to test the model's accuracy. To operationalize the latent constructs from PMT, TCNT and social learning, multiple measurement items are used. The measurement items were adapted from existing literature and edited to fit the needs of this study. Table 3 shows the constructs, measurement items and adaptation.

**Table 3:** Measurement items.

**Construct**	**ID**	**Measurement item**	**Supporting references**
Observation of situation	OSI1	I consider myself well informed regarding the current COVID-19 measures.	van Loenhout et al., 2021
	OSI2	I consider my sources of information regarding COVID-19 credible.	
Threat appraisal	TA1	I am worried about getting sick/infected during my cruise travel.	Zhan et al., 2020
	TA2	I think there is an increased chance that my family will be infected by COVID-19 due to cruise travel.	Coccia, 2020; Nicola et al., 2020; Prasetyo et al., 2020
	TA3	I am worried even if travel restriction is loosened.	Zheng et al., 2021
	TA4	Due to the confined space onboard cruise ships, I perceive higher likelihood of COVID-19 infection.	Prasetyo et al., 2020
	TA5	I am worried about the severe negative impacts on me arising from possible infections.	Chua et al., 2021
	TA6	I think that COVID-19 pandemic outbreak on cruise will be more severe than on land.	Prasetyo et al., 2020
Coping appraisal	CA1	I can adjust my behaviors onboard (e.g.: avoid gathering, always wear masks) and take appropriate measures to ensure the safety of cruise travel.	Wang et al., 2019
	CA2	I already have, or I can learn the necessary skills and acquire equipment to protect myself during my cruise travel.	Zheng et al., 2021
	CA3	I can protect myself well during cruise travel.	Chang, 2021
	CA4	It would take little time or effort for me to protect myself from getting infected while onboard.	Fisher, 2015
Negotiation	NE1	Cruising is important to me that I would like to participate even if there are barriers (e.g., swab test, social distancing).	White, 2008
	NE2	I will persuade myself to engage in cruising, even if the travel constraints still exist.	Loucks-Atkinson and Mannell, 2007
	NE3	I am willing to participate in alternative routes (e.g., Cruise to nowhere) if the desired routes (e.g., Singapore to Bahamas) are not available due to travel constraints.	Kazeminia et al., 2015
Observation of society	OS1	I have seen reports on traditional media regarding cruising activities during the pandemic period.	Park et al., 2020; Lupton and Lewis, 2021; Sharma et al., 2021
	OS2	I have seen articles on online sites and social media regarding cruising activities during the pandemic period.	Channel News Asia, 2020; Orso et al., 2020; Lupton and Lewis, 2021; Sharma et al., 2021
Intention	IN1	I would like to encourage friends/ family to participate in cruising post-COVID-19.	Pan et al., 2021
	IN2	I will take cruise travel whenever I have a chance post-COVID-19.	Zheng et al., 2021
	IN3	Discounts or promotions for cruise travel will further incentivise me to participate in cruise travel.	Lang et al., 2021
	IN4	I have invested or will certainly invest time and money to cruise travel.	Bae and Chang, 2021

The measurement items employ a 7-point Likert scale, on which respondents rate their agreement with the measurement items. A preliminary survey of 51 participants was conducted with the company's assistance to test for any anomalies in results or problematic questions, such as lack of clarity or improper measurements. All measurement items in the threat appraisal construct are negatively phrased, with the exception of TA3, which is changed from “I will feel safer if travel restrictions are relaxed” to “I am worried even if travel restrictions are relaxed.”

The survey is organized as follows. The first section describes the research objective and states that the responses will only be used for research purposes and that their data will be kept anonymous. Subsequently, questions to help respondents build their profiles were included. The inquiries concern their gender, age, highest level of education, employment status and marital status. This is conducted to build a demographic profile of the respondents. The measurement items are presented in the final section.

### Data collection and sampling

To test the model for empirical validation, an online questionnaire was designed. Rakuten, a professional survey company, was used to administrate the online survey. The official questionnaire was conducted from June 15th to 25th, 2021. During this period, a link of the survey invitation was sent to the panel members. Members who receive an invitation can participate in the official survey by clicking on the link. Responses that only partially completed or failed the attention checker questions were excluded from the analysis. Attention checker questions required respondents to select a specific option, implying that they paid attention to the survey, thereby eliminating low-quality responses. A total of 637 responses were received, with only 400 being valid.

The questionnaire was aimed at people living in Singapore as it is one of the few countries that began cruise to nowhere pilots in late 2020. By the time the survey was conducted, enough people in Singapore would have heard about the resumption of cruise activities. We compared the profile summary of our respondents to the Singapore census to ensure proper distribution. Except for employment status and educational background, the distribution of our respondents is largely similar to that of the Singapore Census (National Population Talent Division, 2021). Therefore, it was a fair representation of the general population's consumer mindset. Table 4 shows the profiles and characteristics of our respondents. It is worth noting that the sample of this study included a higher proportion of the elderly population. On the one hand, this is due to the relatively high proportion of the elderly in Singapore's population structure. On the other hand, seniors are a target group in the cruise industry because they tend to have more time and higher consumption levels than the young generation. Therefore, it is necessary for the sample of this study to be more inclusive of the older consumer groups.

**Table 4:** Demographic distribution of respondents.

**Variable**	**Subcategories**	**Percentage (%)**	**Frequency (*n*)**	**Singapore census (locals)**
Age group	18–29	11.0%	44	15.1%
	30–39	19.3%	77	14.8%
	40–49	23.5%	94	15.1%
	50–59	20.5%	82	14.9%
	60 or above	25.8%	103	22.2%
Gender	Male	53.5%	214	48.9%
	Female	46.5%	186	51.1%
Annual household income	SGD 30,000 or less	17.5%	70	19.0%
	SGD 30,001–50,000	13.3%	53	18.0%
	SGD 50,001–70,000	12.8%	51	12.0%
	SGD 70,001–90,000	17.3%	69	13.0%
	SGD 90,001–110,000	13.0%	52	10.0%
	SGD 110,001 or more	26.3%	105	28.0%
Marital status	Married	65.0%	260	58.8%
	Single	34.0%	136	31.5%
Employment status	Full time employed	65.5%	262	65.3%
	Part-time employed	13.8%	55	
	Unemployed but seeking opportunities	7.3%	29	2.8%
	Retired or still in the process of attaining degree	13.5%	54	31.9%
Education background	Secondary and below	13.1%	52	41.8%
	Post-secondary (non-tertiary)	7.8%	31	10.0%
	Diploma and professional qualification	26.5%	106	15.3%
	University	52.8%	211	33.0%

### Data analysis tools

Structural Equation Modeling (SEM) was used to analyze the obtained data. Structural Equation Modeling (SEM) was used to analyze the received data. There are a couple of benefits to adopting SEM. Firstly, SEM is powerful in validating measurement and structural models with latent constructs and complex relationships. Besides, SEM estimation results are more reliable for measurement error is considered (Fornell and Larcker, 1981). Finally, the latent constructs can be estimated by several observable variables (Joseph et al., 2014). As a result, this study adopted this method following the previous research. In the analysis progress, confirmatory Factor Analysis (CFA) is performed first. It determines whether the constructs in the model are properly fit, as well as the reliability and validity of the measurements. Then, SEM was used to test hypotheses. SPSS 21 and AMOS 19 were used as analytical software.

## Results and discussion

### Confirmatory factor analysis

Table 5 summarizes the results of confirmatory factor analysis. It shows the constructs, measurement items, average variance extracted (AVE) and composite reliability (CR). Meanwhile, Table 6 displays a construct matrix with the AVE and squared correlations.

**Table 5:** Confirmatory factor analysis and scale reliability.

**Construct**	**Item**	**λ**	AVE	CR
Observation of situation (OSI)	OSI1	0.811	0.645	0.784
	OSI2	0.789		
Threat appraisal (TA)	TA1	0.764	0.662	0.921
	TA2	0.835		
	TA3	0.866		
	TA4	0.887		
	TA5	0.772		
	TA6	0.748		
Coping appraisal (CA)	CA1	0.676	0.601	0.856
	CA2	0.846		
	CA3	0.877		
	CA4	0.680		
Negotiation (NE)	NE1	0.908	0.732	0.890
	NE2	0.925		
	NE3	0.718		
Observation of Society (OS)	OS1	0.787	0.608	0.757
	OS2	0.773		
Intention (IN)	IN1	0.875	0.655	0.882
	IN2	0.836		
	IN3	0.667		
	IN4	0.842		

*Model fit indices: χ^2^/df = 2.075, (p < 0.05); CFI = 0.965; TLI = 0.958; RMSEA = 0.052; SRMR = 0.045*.

**Table 6:** Average variance extracted and squared correlations of constructs.

	**OSI**	**TA**	**CA**	**NE**	**OS**	**IN**
OSI	0.645	0.007	0.223	0.023	0.370	0.089
TA		0.662	0.044	0.135	0.013	0.101
CA			0.601	0.249	0.12	0.266
NE				0.732	0.114	0.726
OS					0.608	0.178
IN						0.655

*Main diagonal contains average variance extracted values (AVE). Squared correlations between the constructs are above the main diagonal*.

To determine if the model fits well, we used Hu and Bentler (1999) model fit recommendations from Table 5. The results are within the recommended range (χ^2^/df = 2.075, *p* < 0.05; CFI = 0.965; TLI = 0.958; RMSEA = 0.052; SRMR = 0.045), indicating a good model fit.

The model reliability of the measurement items was examined by looking at the factor loading (λ) of each measurement item and the construct CR. For a reliable model, the λs should exceed 0.7 and CR should be <0.8. The reliability test was passed by all of the λs and CRs in Table 5.

Convergent and discriminant analysis are used to test the validity of measurement items. To achieve convergent validity, we must obtain AVE of each construct that is <0.50 (Joseph et al., 2014). Moreover, the AVE of each construct must be larger than the squared correlation of constructs to support the discriminant validity (Fornell and Larcker, 1981). The following criteria are met in Table 6, implying discriminant validity.

### Structural model analysis

**Figure 2:**
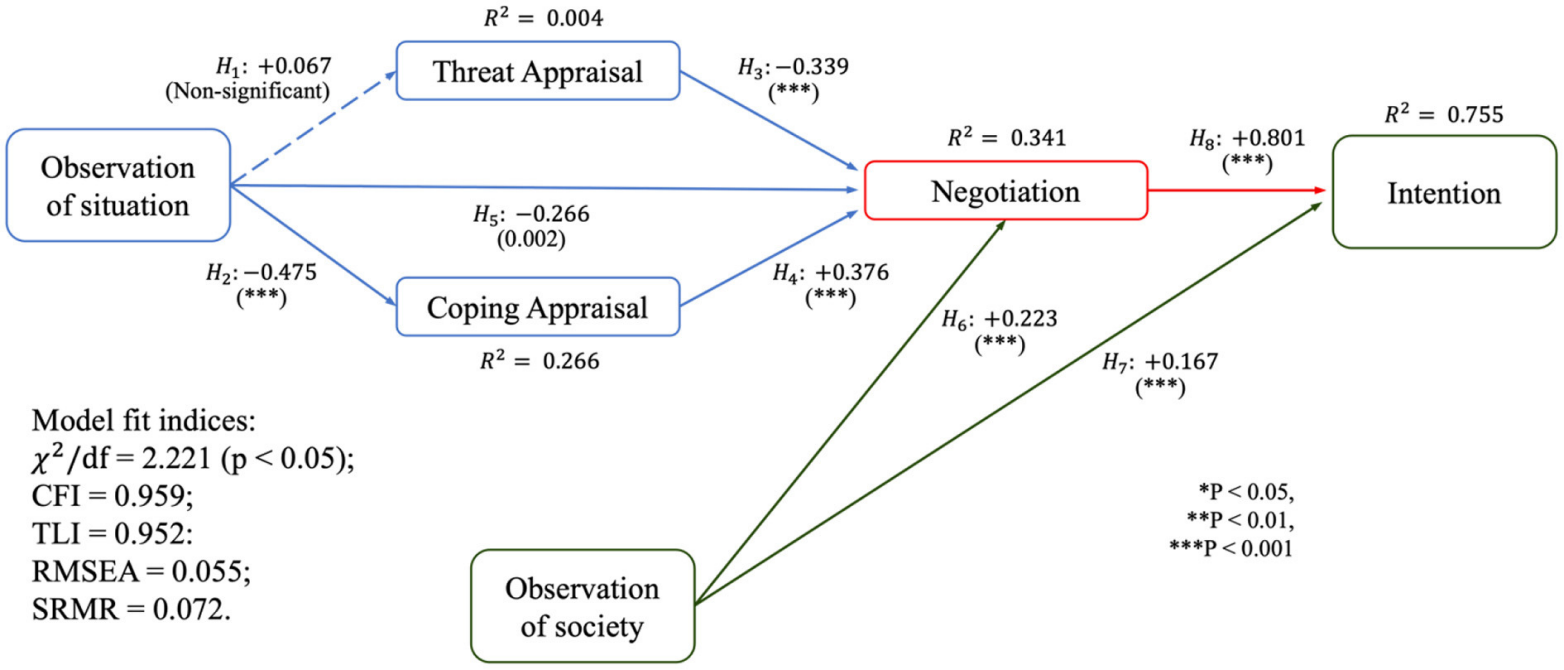
Estimated model.

The SEM results are shown in Figure 2. Except for H1, all of the research hypotheses were significant. Although situation observation influences threat appraisal positively (b = 0.067, *p* > 0.05), the *p*-value renders H1 non-significant. Observation of the situation influences coping appraisal positively (β = −0.475, *p* < 0.001), supporting H2. Meanwhile, threat appraisal has a negative impact on negotiation (β = −0.339, *p* < 0.001), thus supporting H3. Coping appraisal influences negotiation positively (β = 0.376, *p* < 0.001), supporting H4. However, situation observation has a negative influence on negotiation (β = −0.266, *p* < 0.05), supporting H5. Society observation has a positive influence on negotiation (β = 0.223, *p* < 0.001), supporting H6. Moreover, observation of society influences intention positively (β = 0.167, *p* < 0.001), supporting H7. Lastly, negotiation influences intention positively (β = 0.801, *p* < 0.001), thus supporting H8.

For H1, observation of the situation is positively associated with threat appraisal, which makes sense given that greater knowledge of the COVID-19 situation would increase a person's appraisal of the threat. However, H1 was found to be non-significant, which is surprising given that previous studies on health-related issues demonstrated that knowledge of the situation positively and significantly influenced threat appraisal (Xiao et al., 2014). One reason for the non-significance of H1 (Observation of situation → threat appraisal) could be that this survey was conducted in July 2021, 1 year and 4 months after COVID-19 was declared a pandemic (Ducharme, 2020). According to a WHO executive report, pandemic fatigue occurs when people's threat assessment of the virus decreases over time, even though the number of cases of infection may increase (World Health Organization, 2020). People lose motivation to protect themselves from the threat due to prolonged exposure to news and safety measures taken to protect themselves from the virus, perceiving a lower probability of encountering the threat, even if the threat is present and even increasing. Thus, the demotivator influences the threat assessment. PMT is based on the assumption that people are motivated to protect themselves from a threat because they are afraid. However, a drop in motivation may indicate pandemic fatigue, explaining why H1 is non-significant.

Meanwhile, H2 (observation of situation → coping appraisal) is significant and negatively related, which makes sense given that a more difficult COVID-19 situation would impair the individual's ability to deal with the threat. Lahiri et al. (2021) summarized that a more severe COVID-19 threat necessitates more protection by the individual, which may not always be present due to limited ability to protect and higher response costs. This impairs their ability to deal with the threat, lowering their coping appraisal. Similarly, WHO warns that more severe observation will affect the coping appraisal, in which the individual believes the cost of responding outweighs the threat implications (Ahorsu et al., 2020).

H3 (Threat appraisal → negotiation) and H4 (Coping appraisal → negotiation) are theoretically consistent with the theoretical underpinning of TCNT. Increased threat appraisals and decreased coping appraisals increase an individual's constraints. As a result, they must deal with increasing differences in travel expectations and behavior (Kazeminia et al., 2015; Karl et al., 2021), which has a negative impact on negotiation.

H5 (Observation of situation → Negotiation) is significant and has a negative correlation. This implies that observation of COVID-19 severity has a negative impact on negotiation, which is consistent with PMT and TCNT that increased severity causes increased fear, which increases constraints and, according to TCNT, limits negotiation (Karl et al., 2021; Zheng et al., 2021). Because the paths (Observation of situation → Coping appraisal → Negotiation) are significant, coping appraisal serves as a mediator. Meanwhile, because “Observation of situation → Threat appraisal” path is non-significant, threat appraisal can be viewed as an exogenous variable.

H6 (Observation of society → negotiation) and H7 (Observation of society → intention) show significant and positive correlations between the constructs. This is consistent with the theoretical research indicating that SLT influences decision-making (Nabavi, 2012).

Finally, H8 (Negotiation → Intention) is positively and significantly correlated, which aligns with the theoretical study of TCNT, where an increase in negotiation allows travel expectations and behavior to align, hence increasing travel intention. This is consistent with the findings of Karl et al. (2021).

Because H6, H7, and H8 are significant, we can conclude that observation of society has a direct impact on behavioral intention, with negotiation acting as a mediator variable. There is an indirect effect of observation of society → negotiation → intention.

## Conclusion

This study investigated how observing society's behavior and the COVID-19 situation affect people's cruise travel habits. The model's fundamental theory was PMT. It is used to determine how an individual's protection from COVID-19 influences their intention. This is enhanced by the application of SLT to uncover how observing other people in society deal with COVID-19 affects cruise travel behavior. Similarly, travel constraint negotiation theory improves understanding because, despite the constraints, constraint negotiation affects the intention to cruise travel. The hypotheses are empirically tested and demonstrated that COVID-19 protection motivation and societal behavior have a significant impact on an individual's negotiation to cruise travel. The negotiation to travel with constraints will eventually influence the behavioral intention to cruise travel.

Furthermore, the observation of the situation leading to threat assessment was insignificant. A likely deduction pointed to pandemic fatigue, in which long-term protection motivation lowers an individual's perceived threat more than the actual threat.

### Theoretical contributions

Several theoretical contributions were made as a result of this research. On the one hand, by incorporating TCNT and social learning into PMT for post-COVID-19 cruise travel, we yield an examination of behavioral intention that is more holistic, accounting for both individual factual observation and societal behavior observation. To the best of the author's knowledge, there is currently a lack of studies that integrates PMT and social learning, nor with PMT, TCNT and social learning. As a result, this study extends existing research regarding cruise travel intentions in the context of COVID-19.

On the other hand, this study validates the internal relationship between PMT, SLT and TCNT. Specifically, this study emphasized the non-importance of situation observation to threat assessment in protection motivation for the COVID-19 situation. Surprisingly, PMT was not fully applicable this time, and pandemic fatigue was a logical explanation. Pandemic fatigue, which results from prolonged protection from a threat, could eventually lead to a partial change in the PMT model. Thus, it suggests that PMT will be most applicable when the threat is new, people are fearful of it and they are genuinely motivated. The research findings also imply that societal behavior is important in the negotiation of travel constraints and behavioral intention. Although threat and coping appraisal remain, societal behavior aids in managing the gap between travel behavior and travel expectations, thereby influencing travel intentions. Hence, travel constraint negotiation has been shown to have a social component.

### Practical contributions

This research will be useful to the different stakeholders, including cruise lines and government agencies.

To begin, because observation of society is an important determinant of behavioral intention, cruise lines should increase their publicity to encourage increased observation. Subramanian (2018) claimed that traditional advertisement and sales promotion still work, but word-of-mouth, particularly through social media, is more critical. An active presence on social media and traditional media will expose more people to images of people embarking on cruises and participating in cruising activities. They should also encourage and incentivize consumers to post online. Government agencies, particularly the tourism board, can help by promoting through official channels or allowing tourism credits to be used for cruises. Singapore's government has introduced SingapoRediscover Vouchers (Teh and Lim, 2020), which provide credit for citizens to spend in the tourism industry by the end of 2021, but it excludes the cruise industry. With a substantial number of people still not redeeming their SingapoRediscover Vouchers by November 2021 (Subramanian, 2018), the Singapore government should consider including the cruise industry to incentivise spending, thereby assisting the cruise industry's recovery.

Next, protection motivation influences constraint negotiation and, as a result, behavioral intention. Consumers' threat and coping assessments should be reduced by cruise companies. To reduce the perceived threat onboard, safe distancing and COVID-19 restrictions must still be enforced. In the event of an outbreak, medical personnel could be increased, reducing the severity of the risk. Cruise companies should publicize the safety measures taken by the cruise company to assure consumers that they can and are effectively protected against the COVID-19 thread to increase their perceived coping appraisal.

Furthermore, due to pandemic fatigue, the public perceives the threat to be lower than it is. People are less motivated to protect themselves when they perceive a lower threat. One of the WHO recommendations is to limit restrictions that reduce risk while having a minimal impact on daily activities (World Health Organization, 2020). While tightening restrictions is understandable, the public is generally judging the threat incorrectly based on factual observations. Consumers in the cruise industry may begin to disregard regulations if they believe the threat is low. Cruise companies will have to ensure that passengers continue to follow the regulations, which may necessitate more staff on board to remind consumers to wear masks and maintain safe distance. Otherwise, the actual risk of a COVID-19 outbreak rises, and operations are disrupted once more.

#### Limitations and future research

This study has several limitations, but it does provide opportunities for future research. For starters, the social learning aspect of this study was not thoroughly investigated. Although social learning has been demonstrated to be related and to have an impact on decision-making, it can still be broken down into smaller constructs to investigate the various aspects of social learning in PMT.

Next, the COVID-19 situation is constantly changing, influencing public expectations. Another area of investigation would be the effect of time, the number of cases and the motivation for protection. As COVID-19 cases arrive in waves, protection motivation may not decrease linearly over time. It would be useful to learn how motivation changes over time and in light of the constantly changing COVID-19 situation.

## Data availability statement

The raw data supporting the conclusions of this article will be made available by the authors, without undue reservation.

## Ethics statement

Ethical review and approval was not required for the study on human participants in accordance with the local legislation and institutional requirements. The patients/participants provided their written informed consent to participate in this study.

## Author contributions

KY: conceptualization, survey design, data collection, writing, and revision. XW: writing, editing, and revision. All authors contributed to the article and approved the submitted version.

## Funding

This research was supported by the Chung-Ang University Research Grants in 2020.

## Conflict of interest

The authors declare that the research was conducted in the absence of any commercial or financial relationships that could be construed as a potential conflict of interest.

## Publisher's note

All claims expressed in this article are solely those of the authors and do not necessarily represent those of their affiliated organizations, or those of the publisher, the editors and the reviewers. Any product that may be evaluated in this article, or claim that may be made by its manufacturer, is not guaranteed or endorsed by the publisher.
